# Monitoring of argatroban and lepirudin anticoagulation in critically ill patients by conventional laboratory parameters and rotational thromboelastometry – a prospectively controlled randomized double-blind clinical trial

**DOI:** 10.1186/s12871-018-0475-y

**Published:** 2018-02-09

**Authors:** Martin Beiderlinden, Patrick Werner, Astrid Bahlmann, Johann Kemper, Tobias Brezina, Maximilian Schäfer, Klaus Görlinger, Holger Seidel, Peter Kienbaum, Tanja A. Treschan

**Affiliations:** 1 Klinik für Anästhesiologie, Marienhospital Osnabrück, Bischofsstr. 1, 49076 Osnabrück, Germany; 2 Klinik für Anästhesiologie, Universitätsklinik Düsseldorf, Heinrich-Heine-Universität Düsseldorf, Moorenstr. 5, 40225 Düsseldorf, Germany; 3 TEM International GmbH, Martin-Kollar-Str. 13-15, 81829 Munich, Germany; 4 Institut für Hämostaseologie, Hämotherapie und Transufsionsmedizin, Universitätsklinik Düsseldorf, Heinrich-Heine-Universität Düsseldorf, Moorenstr. 5, 40225 Düsseldorf, Germany

**Keywords:** Heparin-induced thrombocytopenia, Rotational thromboelastometry, Argatroban, Lepirudin, Monitoring, Maximum clot firmness

## Abstract

**Background:**

Argatroban or lepirudin anticoagulation therapy in patients with heparin induced thrombocytopenia (HIT) or HIT suspect is typically monitored using the activated partial thromboplastin time (aPTT). Although aPTT correlates well with plasma levels of argatroban and lepirudin in healthy volunteers, it might not be the method of choice in critically ill patients. However, in-vivo data is lacking for this patient population.

Therefore, we studied in vivo whether ROTEM or global clotting times would provide an alternative for monitoring the anticoagulant intensity effects in critically ill patients.

**Methods:**

This study was part of the double-blind randomized trial “Argatroban versus Lepirudin in critically ill patients (ALicia)”, which compared critically ill patients treated with argatroban or lepirudin. Following institutional review board approval and written informed consent, for this sub-study blood of 35 critically ill patients was analysed. Before as well as 12, 24, 48 and 72 h after initiation of argatroban or lepirudin infusion, blood was analysed for aPTT, aPTT ratios, thrombin time (TT), INTEM CT,INTEM CT ratios, EXTEM CT, EXTEM CT ratios and maximum clot firmness (MCF) and correlated with the corresponding plasma concentrations of the direct thrombin inhibitor.

**Results:**

To reach a target aPTT of 1.5 to 2 times baseline, median [IQR] plasma concentrations of 0.35 [0.01–1.2] μg/ml argatroban and 0.17 [0.1–0.32] μg/ml lepirudin were required. For both drugs, there was no significant correlation between aPTT and aPTT ratios and plasma concentrations. INTEM CT, INTEM CT ratios, EXTEM CT, EXTEM CT ratios, TT and TT ratios correlated significantly with plasma concentrations of both drugs. Additionally, agreement between argatroban plasma levels and EXTEM CT and EXTEM CT ratios were superior to agreement between argatroban plasma levels and aPTT in the Bland Altman analysis. MCF remained unchanged during therapy with both drugs.

**Conclusion:**

In critically ill patients, TT and ROTEM parameters may provide better correlation to argatroban and lepirudin plasma concentrations than aPTT.

**Trial registration:**

ClinicalTrials.gov, NCT00798525, registered on 25 Nov 2008

**Electronic supplementary material:**

The online version of this article (10.1186/s12871-018-0475-y) contains supplementary material, which is available to authorized users.

## Background

Argatroban and lepirudin are direct thrombin inhibitors, approved for alternative anticoagulation of patients with proven or suspected heparin induced thrombocytopenia.

Argatroban, a parenteral recombinant L-arginine derivate, is primarily metabolized in the liver. In contrast, lepirudin, a parenteral recombinant hirudin, is eliminated primarily through the kidneys. Thus, argatroban has to be carefully titrated in patients with reduced liver function. Lepirudin needs to be carefully adapted to those with reduced kidney function. Especially in critically ill patients, the appropriate dosage of both drugs has been shown to be very challenging [1, 2].

For argatroban we have shown that the initially recommended dose of 2 μg/kg/min is far too high for critically ill patients [3]. This finding was enhanced by Link and co-workers, who demonstrated an inverse correlation between the severity scores SAPS II and SOFA and argatroban dosing requirements, i.e. the sicker the patient and the higher the SAPS II or SOFA, the less argatroban is required to reach the target activated partial thromboplastin time (aPTT) [4, 5]. As a consequence, the manufacturer currently recommends a low argatroban starting dose of 0.5 μg/kg/min for critically ill patients and for patients with hepatic insufficiency as defined by a Child-Pugh Class B. However, many critically ill patients show, if at all, only moderately elevated hepatic laboratory variables and do not fulfil the Child-Pugh criteria for hepatic impairment and require even lower argatroban starting doses [6].

Similarly, lepirudin is recommended to be administered using a bolus dose of 0.4 mg/kg body weight plus a continuous infusion of 0.15 mg/kg/h [1]. However, this has also been shown to be too high and it has been proven that omitting the bolus and reducing the continuous infusion to 0.08 mg/kg/h in patients without renal failure and to less than 0.04 mg/kg/h in patients with renal failure results in 1.5 to 2 times baseline aPTT values [1].

For routine use, aPTT is recommended for monitoring the anticoagulatory effect of argatroban and lepirudin, as aPTT correlates well enough with the plasma concentrations of both drugs in healthy volunteers [7, 8].

However, in critically ill patients, aPTT might not be the ideal parameter to monitor argatroban and lepirudin. First, aPTT might be prolonged in critically ill patients even without anticoagulant medication, due to variable factors, often activation of the contact phase system (by the use of extracorporeal devices), a (bleeding associated) deficit of coagulation factors or rarely appearance of a lupus anticoagulant [9]. Secondly, very low doses of direct thrombin inhibitors often result in prolonged aPTT, but it remains unknown, whether these aPTT values correspond to appropriate plasma concentrations of direct thrombin inhibitors.

Therefore, the question of additional monitoring options for direct thrombin inhibitors in critically ill patients needs to be addressed. A potentially useful addition to aPTT measurements could result from the evaluation of coagulation by rotational thromboelastometry (ROTEM). ROTEM allows a rapid and bedside visualisation and quantification of coagulation parameters. It has been proven beneficial as part of goal-directed bleeding management in patients with major haemorrhage [10–12]. ROTEM measures clot formation in different assays, each specifically depicting different coagulation pathways. The INTEM assay models intrinsic coagulation activation, the pathway conventionally assessed by aPTT measurements. The EXTEM assay models extrinsic coagulation activation, which usually is reflected by prothrombin time (PT, German: “Quick”) measurements. During the FIBTEM assay, platelets are inactivated, which results in a qualitative evaluation of the fibrinogen status of the coagulation system. This step is partially reflected by measurement of the thrombin time (TT), which is typically referred to by the historic “common pathway of coagulation”.

Conflicting results have been reported on the ability of ROTEM to monitor argatroban and lepirudin effects in vitro [13, 14]. To our knowledge, so far no information is available on the results of ROTEM in critically ill patients treated with argatroban or lepirudin.

Therefore, we aimed to study whether ROTEM could be useful for monitoring the anticoagulant effect of argatroban or lepirudin in critically ill patients. As a pre-planned sub-study of the recently published larger double-blinded clinical trial (“Argatroban versus Lepirudin in critically ill patients (ALicia”) [15], we evaluated the results of ROTEM measurements and conventional laboratory parameters for monitoring anticoagulation and tested their correlation to drug plasma levels in critically ill patients undergoing argatroban and lepirudin therapy.

## Methods

The Ethics committee of the Medical Faculty, Heinrich-Heine-University Düsseldorf, Germany and the Bundesinstitut für Arzneimittel, EudraCT number 2006–003122-28 approved this study (ClinicalTrials.gov number NCT00798525, registered 25th of November 2008), which has been performed in accordance with the Declaration of Helsinki in its effective form. This study was part of the trial “Argatroban versus Lepirudin in critically ill patients (ALicia)” [15]. Details of in- and exclusion criteria are specified elsewhere [15]. In short, critically ill patients with suspected HIT were treated with argatroban (the argatroban-group) or lepirudin (the lepirudin-group), aiming at a target aPTT of 1.5 to 2 times baseline. We included surgical intensive care unit patients with expected ICU treatment > 24 h, age ≥ 18 years and suspected HIT (decrease in platelet count > 50% from baseline, persisting for more than 24 h, 4 T-Score > 3 or positive PF4/heparin enzyme-linked immunosorbent assay). Randomisation was based on a computer generated multi-block 1:1 group assignment. Written informed consent was obtained from patients or their legal guardian. Between February 2010 and November 2011, blood samples were analysed for this sub-study.

### Dosage and application of study drugs

In the argatroban group, patients without liver dysfunction received an intravenous starting dose of 0.5 μg/kg/min. Patients with liver dysfunction, defined as bilirubin > 4 mg/dl, received 0.25 μg/kg/min. In the lepirudin group, patients without renal impairment were treated with a starting dose of 0.05 mg/kg/h, patients with moderate renal impairment received 0.01 mg/kg/h and patients with continuous renal replacement therapy 0.005 mg/kg/h. Study drugs were prepared by personnel not involved in data collection and delivered to the ward and applied in neutral syringes to facilitate blinding of patients and personnel involved in treatment.

### Blood samples and measurements

Blood samples for immediate ROTEM analyses were collected in sodium citrated tubes. These samples were taken at baseline, defined as prior to start of alternative anticoagulation, and at the following time points: 12, 24, 48 and 72 h after start of alternative anticoagulation. Simultaneously, blood was sent to the institutional laboratory for an analysis of the conventional coagulation variables aPTT, PT and TT. aPTT (Pathromtin SL®), PT (Thromborel S®) and TT (BC® Thrombin) were analysed on Behring Coagulation System BCS Analyzer (Siemens, Marburg, Germany). Samples for argatroban and lepirudin plasma concentrations were centrifuged, pipetted and stored at − 80 °C for subsequent batch analyses. ECA (Diagnostica Stago, Asnieres, France) was adapted to the BCS Analyzer.

A ROTEM Thromboelastometer (TEM Innovation GmbH, Munich, Germany) was used for measurement of INTEM, EXTEM and FIBTEM. According to the manufacturer’s instructions, all measurements were performed at a temperature of 37 °C, using the automated pipetting tools of the device. According to the manufacturer’s instructions, for each assay 300 μl of decalcified whole blood had been recalcified with the manufacturer’s star-tem®solution plus addition of in-tem® (partial thromboplastin, ellag acid, and buffer solution) or ex-tem®(recombinant tissue factor, phospholipids, heparin-inhibitor and buffer solution), respectively. For FIBTEM assays, ex-tem® and fib-tem® (cytochalasin and buffersolution) were used.

The following variables of ROTEM monitoring were analysed: Clotting time (CT): time in seconds from start of the analysis until start of clot formation. Maximum clot firmness (MCF): maximum amplitude in millimetres.

CT and CT ratios, the quotient of each measurement and the corresponding baseline CT, were evaluated for INTEM and EXTEM. MCF during treatment with argatroban or lepirudin was evaluated by INTEM, EXTEM and FIBTEM.

Results of ROTEM measurements, for which automatically generated failure codes occurred, were sent to the TEM®-Support department (TEM Innovation GmbH, Munich, Germany) for evaluation and only analysed further if assessed as valid.

### Correlations

Correlations between monitoring parameters and argatroban and lepirudin plasma levels were graphically displayed and correlation coefficients calculated for aPTT, aPTT ratios, which are the quotient of every aPTT and the corresponding baseline value, PT (Quick), TT and TT ratios, INTEM CT, INTEM CT ratios, EXTEM CT and EXTEM CT.

### Bland Altman plots

Bland Altman analysis was performed for agreement between argatroban plasma levels and aPTT, EXTEM CT and EXTEM CT ratios.

### Statistics

Using SPSS Statistics 22 (IBM, New York, USA), the data were tested for normal distribution with the KS-test. Continuous variables were compared with t-test, Mann-Whitney-U-test or Wilocoxon, as appropriate. The data are displayed as mean and standard deviation or median and quartiles.

### Sample size

This study was purely exploratory, thus no prospective sample size calculation had been done.

### Level of significance

For univariate comparison between groups, a two-sided *p* value < 0.05 was considered to be statistically significant. To allow for comparison between groups over time, a Bonferroni corrected *p*-value < 0.01 was requested. To account for multiple comparisons within a group, a p-value < 0.0125 was considered statistically significant. Similarly, when employing Spearman correlation, statistical significance was indicated by *p*-values < 0.01, due to multiple comparisons.

## Results

Blood of 35 patients was analysed, of whom 17 had been treated with argatroban and 18 with lepirudin. The biometric and laboratory variables upon admission to the intensive care unit are depicted in Table 1. Coagulation parameters at baseline where within the normal range except for aPTT, which was slightly elevated above the upper limit of 37 s in both groups after heparin infusion had been stopped. There were no significant differences between patients in the argatroban- or lepirudin-group, except for higher calcium values in the lepirudin-group (Table 1).

**Table 1:** Patient characteristics

	Argatroban (*n* = 17)	Lepirudin (*n* = 18)	*p*
Gender (male/female)	12/5	10/8	0.463
Age (years)	72 ± 10	59 ± 18	0.053
Height (cm)	172 ± 10	171 ± 8	0.732
Weight (kg)	89 ± 19	76 ± 19	0.067
Body mass index	30 ± 9	26 ± 6	0.126
Systolic blood pressure (mmHg)	123 ± 18	122 ± 21	0.684
Diastolic blood pressure (mmHg)	57 ± 10	58 ± 11	0.757
Mean arterial blood pressure (mmHg)	81 ± 14	76 ± 13	0.443
Heart beats per minute	93 ± 19	93 ± 18	0.883
Glasgow coma scale	8 ± 5	6 ± 4	0.232
Simplified Acute Physiology Score	38 ± 16	35 ± 15	0.732
Sequential Organ Failure Assessment Score	11 ± 7	10 ± 4	0.636
aPTT (seconds)	47 ± 8	45 ± 9	0.732
Quick (%)	79 ± 24	81 ± 16	0.832
INR	1.2 ± 0.3	1.2 ± 0.3	0.807
TZ (seconds)	30 ± 26	20 ± 7	0.568
Fibrinogen (mg/dl)	705 ± 242	721 ± 159	0.851
Antithrombin (%)	79 ± 22	83 ± 20	0.467
Leukocyts (/nl)	11.9 ± 3.8	15.6 ± 11	0.684
Red blood cell count (/pl)	3.4 ± 0.3	3.2 ± 0.3	0.184
Hemoglobin (g/dl)	9.8 ± 0.9	9.4 ± 0.9	0.219
Hematocrit (%)	31 ± 3	29 ± 3	0.077
Thrombocytes (/nl)	130 ± 106	189 ± 198	0.386
Sodium (mmol/l)	142 ± 5	141 ± 4.	0.763
Potassium (mmol/l)	4.4 ± 0.4	4.5 ± 0.4	0.363
Calcium (mmol/l)	1.17 ± 0.05	1.27 ± 0.24	0.026*
Creatinine (mg/dl)	1.9 ± 1.2	1.8 ± 1.2	0.658
Urea (mg/dl)	89 ± 46	93 ± 48	0.865
Total protein (g/dl)	5.5 ± 0.3	5.7 ± 0.3	0.299
Albumin (g/dl)	2.5 ± 0.4	2.4 ± 0.4	0.503
Bilirubin total(mg/dl)	1.8 ± 2.7	5.8 ± 10.4	0.118
GOT (AST; U/l)	307 ± 547	273 ± 642	0.606
GPT (ALT; U/l)	189 ± 225	156 ± 303	0.145
γ-GT (U/l)	196 ± 192	243 ± 241	0.497
LDH (U/l)	430 ± 176	469 ± 167	0.401
CK total (U/l)	341 ± 830	946 ± 2158	0.161
CK-MB (U/l)	18.8 ± 19.1	38.6 ± 34.4	0.310
Troponin T (ng/ml)	1.11 ± 1.81	0.51 ± 0.81	0.841
CRP (mg/dl)	16 ± 8	18 ± 9	0.381
Glucose (mg/dl)	124 ± 27	135 ± 22	0.259

Data are median ± standard deviation.*p < 0.05

### Plasma concentrations

**Fig. 1:**
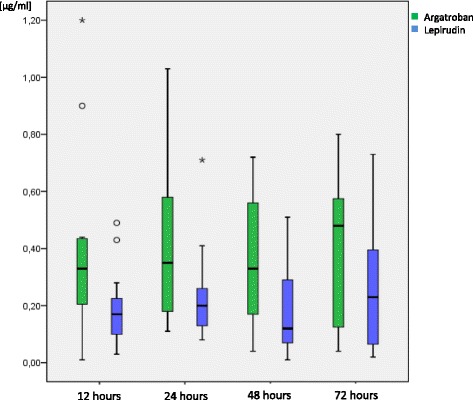
Plasma concentrations of argatroban and lepirudin over time. Data are depicted as boxes showing median and interquartile range, whiskers indicate 1.5 times interquartile range. Outliers are depicted as circles and stars

The plasma concentrations of argatroban and lepirudin over time are depicted in Fig. 1. The mean plasma concentration in the argatroban group was 0.35 [0.01–1.2] μg/ml and thus significantly higher than the mean lepirudin plasma concentration of 0.17 [0.1–0.32] μg/ml (*p* = 0.025).

### Global coagulation parameters

#### aPTT

**Fig. 2:**
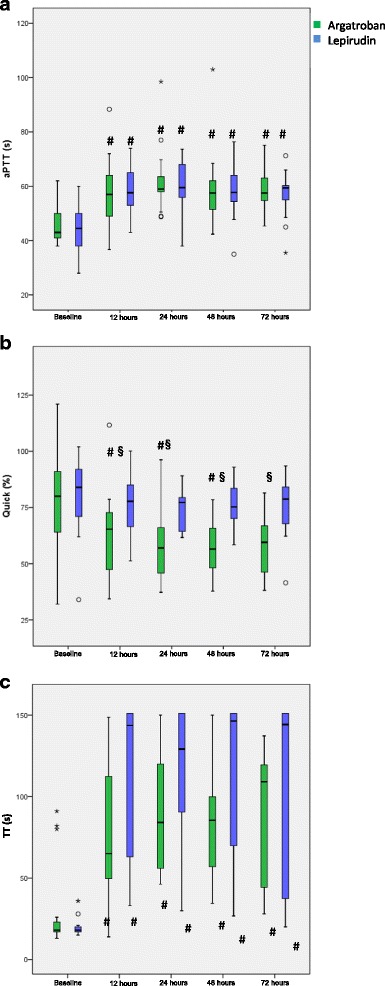
Conventional laboratory coagulation parameters over time. In all panels data are depicted as boxes showing median and interquartile range, whiskers indicate 1.5 times interquartile range. Outliers are depicted as circles and stars. # significant difference to baseline measurements (*p* < 0.0125), § significant differences between groups (p < 0.01): (**a**) aPTT, (**b**) PT, “Quick”, (**c**)TT

Prior to treatment with argatroban or lepirudin, aPTT was comparable between the two groups (Fig. 2a). During treatment, aPTT increased significantly compared to baseline in both groups and remained comparable between patients in the argatroban- and lepirudin-group (Fig. 2 a).

#### PT, “quick”

Prior to treatment, PT was comparable between the argatroban- and lepirudin group. During treatment, PT decreased significantly as compared to baseline only in patients treated with argatroban. Thus, PT during treatment was significantly lower in the argatroban-than in the lepirudin-group (Fig. 2b).

#### TT

TT was comparable between the two treatment groups at baseline and increased significantly during treatment in both groups (Fig. 2c). TT values during treatment were comparable between patients in both groups. In the lepirudin group, 30 measurements were above the upper limit of 150 s during treatment, while in the argatroban group this was only the case for two measurement.

#### ROTEM measurements

**Fig. 3:**
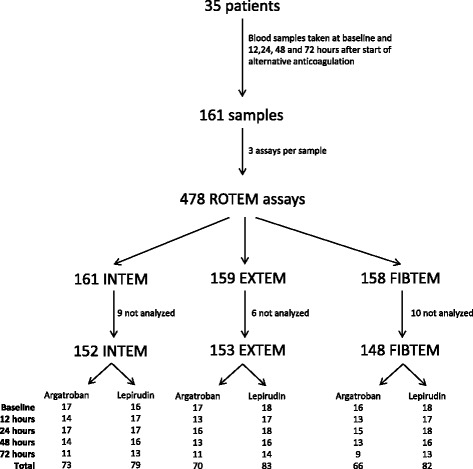
Flow chart of ROTEM measurement. Data are numbers of patients or ROTEM samples. ROTEM measurements were assessed as invalid and results not used for analysis in 25 out of 478 cases (5%) due to automatically generated failure codes such as early drying of the sample or lack of measureable coagulation

Blood samples of all patients yielded a total of 478 ROTEM measurements, 25 of which were assessed as invalid, resulting in 453 assays for analysis (Fig. 3). The main reason for invalid measurements were early drying of the sample and missing deflection in the TEMogramm.

#### INTEM ct

**Fig. 4:**
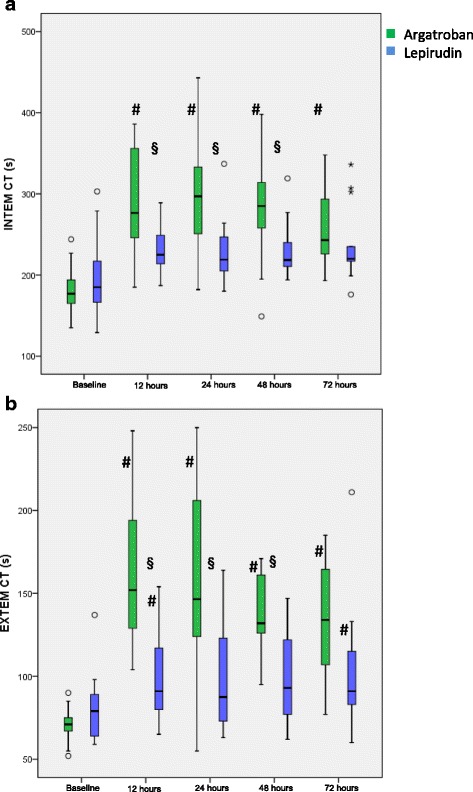
Clotting time measured by INTEM and EXTEM over time. In all panels data are depicted as boxes showing median and interquartile range, whiskers indicate 1.5 times interquartile range. Outliers are depicted as circles and stars. # significant difference to baseline measurements (*p* < 0.0125), § significant differences between groups (*p* < 0.01): (**a**) INTEM CT, (**b**) EXTEM CT

The INTEM CT was comparable between the argatroban and lepirudin group prior to treatment. In the argatroban group, INTEM CT increased significantly compared to baseline. The increase in INTEM CT in the lepirudin group was smaller and statistically not significant. Thus, INTEM CT during treatment was significantly higher in the argatroban-group at 12, 24 and 48 h (Fig. 4a).

#### EXTEM CTct

The EXTEM CT was comparable between the two groups prior to treatment. In the both groups, EXTEM CT increased significantly compared to baseline, with a significantly higher increase in the argatroban-group compared to the lepirudin-group (Fig. 4b).

#### Correlations

##### Argatroban-group

**Fig. 5:**
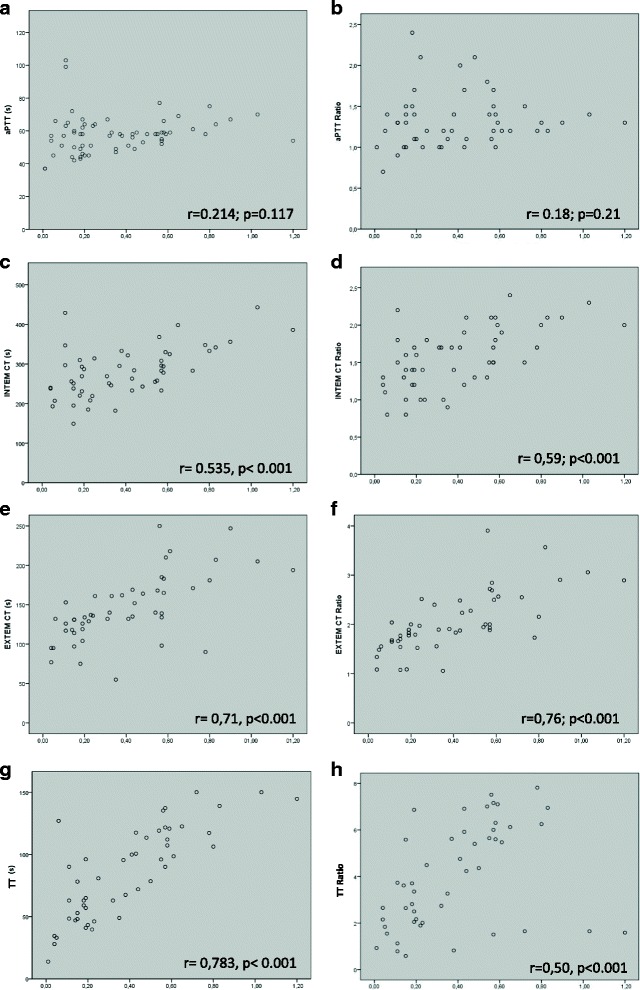
Correlation between conventional coagulation parameters, ROTEM parameters and argatroban plasma levels. X-axis depicts argatroban plasma concentration in μg/ml, Y-axis displays unit of specific coagulation parameter: **a**) aPTT, **b**) aPTT ratio, **c**) INTEM CT, **d**) INTEM CT ratio, **e**) EXTEM CT, **f**) EXTEM CT ratio, **g**) TT and **h**) TT ratio. s = seconds. Each dot represents one pair of measurements. Spearman correlation coefficient (r) and level of significance are presented in each panel, *p* < 0.01 was considered statistically significant

Correlations in patients treated with argatroban are depicted in Fig. 5. We found no correlation between aPTT or aPTT ratios and the argatroban plasma concentrations (Fig. 5 a, b). In contrast, INTEM CT and INTEM CT ratios showed a positive linear correlation of intermediate strength with argatroban plasma concentrations (Fig. 5 c, d). EXTEM CT and EXTEM CT ratios (Fig. 5 e, f) and TT and TT ratios (Fig. 5 g, h) correlated significantly with argatroban plasma concentrations. Quick did not correlate with argatroban plasma levels (Additional file 1).

##### Bland Altman plots

**Fig. 6:**
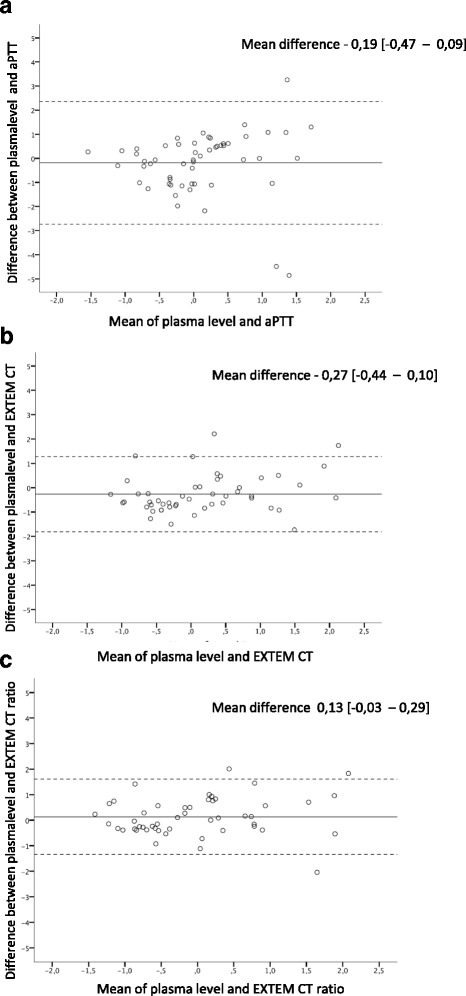
Bland Altman plots for agreement between argatroban plasma levels and aPTT (**a**), EXTEM CT (**b**) and EXTEM CT (**c**) ratios. X-axis depicts the mean between two parameters, Y-axis displays the difference between two parameters. The lowest difference between means is displayed for EXTEM CT ratios

For parameters with significant and strong correlations, i.e. EXTEM CT and EXTEM CT ratios, bland altman plots showed better agreement with argatroban plasma levels, than between aPTT and argatroban plasma levels (Fig. 6 a-c).

##### Lepirudin-group

**Fig. 7:**
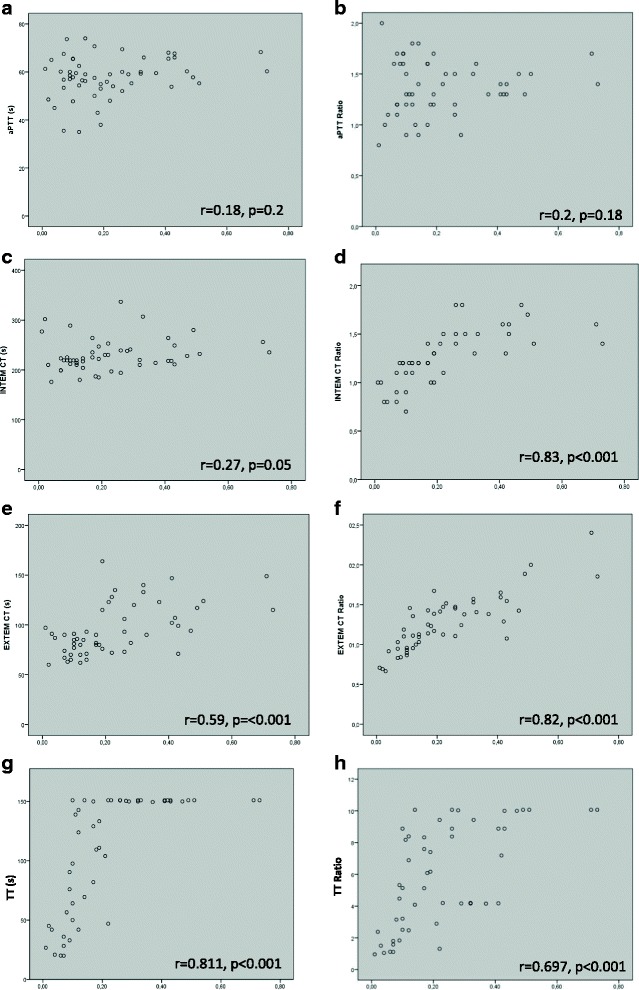
Correlation between conventional coagulation parameters, ROTEM parameters and lepirudin plasma levels. X-axis depicts lepirudin plasma concentration in μg/ml, Y-axis displays unit of specific coagulation parameter. **a**) aPTT, **b**) aPTT Ratio, **c**) INTEM CT, **d**) INTEM CT Ratio, **e**) EXTEM CT, **f**) EXTEM CT Ratio, **g**) TT, **h**) TT Ratio; s = seconds. Each dot represents one pair of measurements. Spearman correlation coefficient (r) and level of significance are presented in each panel, *p* < 0.01 was considered statistically significant

In patients treated with lepirudin we found no significant correlation between the lepirudin plasma concentration and aPTT, aPTT ratio or INTEM CT (Fig. 7 a-c). INTEM CT ratios, EXTEM CT and EXTEM CT ratios and TT correlated significantly with the lepirudin plasma concentrations (Fig. 7 d-h). Quick did not correlate with lepirudin plasma concentrations (Additional file 1).

##### Maximum clot firmness

**Fig. 8:**
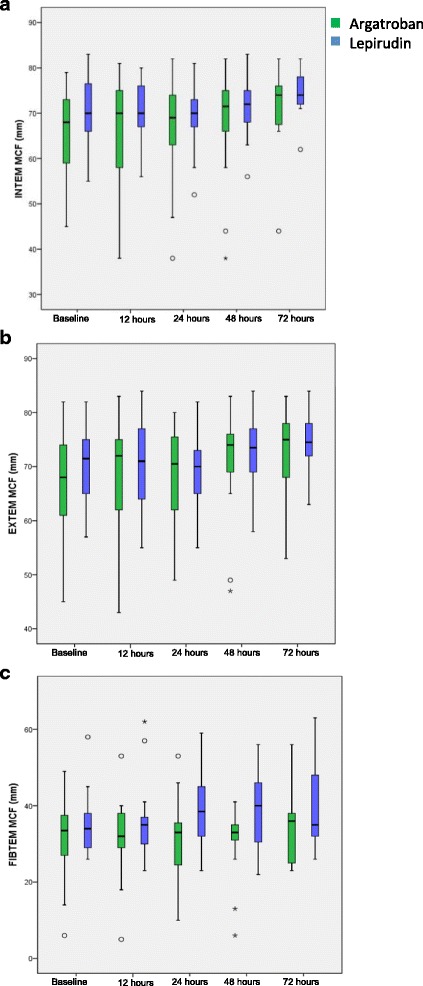
Maximum clot firmness measured by INTEM, EXTEM and FIBTEM over time. In all panels data are depicted as boxes showing median and interquartile range, whiskers indicate 1.5 times interquartile range. Outliers are depicted as circles and stars. There were no statistically significant differences within the argatroban or lepirudin group as compared to baseline or between the argatroban and lepirudin group: **a**) INTEM MCF, **b**) EXTEM MCF, **c**) FIBTEM MCF

The maximum clot firmness, as measured by INTEM, EXTEM and FIBTEM, was similar between patients treated with argatroban or lepirudin prior to and during treatment (Fig. 8 a-c).

## Discussion

To our knowledge this is the first in vivo study on critically ill patients treated with argatroban or lepirudin as alternative anticoagulation that shows that the aPTT does not correlate with plasma levels of both direct thrombin inhibitors. In contrast, parameters measured by ROTEM, specifically EXTEM CT and EXTEM CT ratios, correlate well with the plasma levels of argatroban and lepirudin. Similarly important, of the conventional laboratory coagulation variables, the “classical” TT shows a strong correlation to the plasma concentrations of argatroban and lepirudin in critically ill patients.

Argatroban is and lepirudin was licenced for anticoagulation of patients with HIT and HIT suspect. A target aPTT span of 1.5–3 times the baseline value is recommended for alternative therapeutic anticoagulation with argatroban and lepirudin [7, 16]. Although lepirudin has been taken off the market [17], we think it is worthwhile to present and discuss the results of the lepirudin-group here, for two main reasons: 1) it would be unethical with respect to the participants of our trial not to provide the results gained during this study, 2) results from lepirudin might help to gain insights into mechanisms applicable to other substances which are still available.

Nevertheless, with regard to current practice, parts of the discussion will focus on argatroban, as this drug is now widely used in patients with HIT or HIT suspect.

The European consensus conference on the use of argatroban recommends aPTT for monitoring the drug’s anti-coagulatory effects [18]. However, these recommendations are based on studies in non-critically ill patients or healthy volunteers [19]. The coagulatory system during severe illness has been characterized as being highly susceptible to argatroban, because a tenfold lower dose is sufficient to achieve target aPTTs in critically ill patients [3]. Link et co-workers showed an inverse correlation between severity of illness measured by SAPS and the argatroban dose required to achieve target aPTT in critically ill patients requiring continuous renal replacement therapy [4]. They presumed an unapparent hepatic dysfunction, one undetected by common laboratory variables. This seems reasonable, because indocyanine green plasma disappearance rate correlates well with the argatroban doses necessary to reach the predefined aPTT targets [4].

In the double-blinded randomized setting of our clinical trial, a mean dose of only 0.33 μg/kg/min argatroban was required to achieve the aPTT target of 1.5–2 times baseline in critically ill patients with HIT suspect and need for CRRT [15].

Although, the reason for high susceptibility of critically ill patients to argatroban still remains unclear, albumin plasma concentrations might play an important role [20]. Argatroban is partially bound to albumin and albumin plasma concentrations typically are reduced in critically ill patients [20]. Albumin plasma concentrations were low in the patients we studied (Table 1). Reduced albumin plasma concentration might thus have contributed to higher circulating concentrations of argatroban in these critically ill patients.

Results of several trials indicate that monitoring argatroban by aPTT may not be appropriate in the specific patient population of critically ill [21–23]. One reason is the high inter-assay variability, which means, that results of aPTT measurements differ greatly depending on the reagent used for analysis [22]. Specifically in critically ill patients, variability is increased due to several factors that can lead to over- or underestimation of drug effects, such as low levels of clotting factors or elevated levels of factor VIII [24, 25]. Therefore, the question of a routinely available, easy to perform and cost-effective alternative needs to be addressed. Thrombin time has been shown to correlate better with argatroban and lepirudin concentrations than aPTT [22, 26, 27]. In non-critically ill patients, TT needs to be diluted, in order to remain within the measurable range [26].

Our data add to these findings. In our critically ill patients, we found no correlation between aPTT and argatroban or lepirudin plasma levels. Of note, so far there is no recommendation for a plasma level, that should be targeted in order to provide adequate concentrations of direct thrombin inhibitors [22]. Furthermore, in our “Argatroban versus Lepirudin in critically ill patients (ALicia)” trial, the life-time of filters for continuous renal replacement therapy was rather short, despite aPTT within the range of 1.5–2 times baseline [15]. This might indicate that aPTT results overestimated the anticoagulatory potency of both argatroban and lepirudin. In contrast, TT correlated significantly with argatroban and lepirudin plasma levels. However, results of several samples of the lepirudin-groups reached the upper limit of our routine TT measurements duration of 150 s. Thus, a diluted TT might improve the accuracy of results, specifically for patients treated with lepirudin. In summary, in critically ill patients TT or diluted TT seems to be the parameter of choice to allow monitoring of the anticoagulatory effect of direct thrombin inhibitors that relates to plasma level of argatroban or lepirudin.

According to our findings, ROTEM should also be considered as an option for monitoring the anticoagulatory effects of argatroban and lepirudin. To our knowledge our results presented here are the first to show that EXTEM clotting time and the EXTEM CT ratios correlate well with argatroban and lepirudin plasma concentrations in critically ill patients. For argatroban INTEM CT and INTEM CT ratios also show a significant correlation to plasma concentrations.

The correlation of INTEM CT with argatroban plasma concentrations has been shown ex-vivo in spiked blood of healthy volunteers [13]. It remained unclear whether lower argatroban concentrations might be detected with INTEM CT or EXTEM CT in vivo [28]. Therefore, alternative ROTEM essays, like ECATEM, an ecarin-activated modified TEM assays, have recently been proposed for monitoring argatroban [14]. However, this essay was not available at the time of our study. Therefore, we used two unmodified ROTEM essays for INTEM and EXTEM according to the manufacturer’s guidelines to gain insight into the properties of ROTEM to depict anticoagulatory potency of argatroban and lepirudin in our patients.

INTEM CT and EXTEM CT correlated significantly with the low argatroban plasma concentrations. However, the strength of these correlations was only moderate. The strength of the correlation was increased when INTEM CT and EXTEM CT ratios were used. However, these ratios need additional calculations, which might be an obstacle against the routine use of these parameters on an intensive care unit.

Therefore, further studies are needed to identify the full potential of ROTEM measurements to monitor argatroban anticoagulatory effect in critically ill patients.

Of note, neither argatroban nor lepirudin influenced maximum clot firmness. Maximum clot firmness has been shown to be a significant predictor of bleeding in critically ill patients [29]. In vitro studies had conflicting results indicating that the maximum clot firmness might be reduced or unaffected during anticoagulation with direct thrombin inhibitors [30, 31]. Our results confirm that MCF is preserved in critically ill patients. Thus, reduced clot firmness does not contribute to an increased bleeding risk during treatment with argatroban or lepirudin in critically ill patients.

### Limitations

We studied a small sample size and a quite narrow range of argatroban and lepirudin plasma concentrations, due to the severe critical illness of our patient population. More data on an alternative routine monitoring of argatroban therapy in critically ill patients are needed. Our data add to the cumulating evidence that aPTT is not the appropriate monitoring tool for critically ill patients and that TT or diluted TT should be taken into account as an alternative. Furthermore, our data indicate, that ROTEM parameters have the potential to supplement conventional anti-coagulation monitoring. Nevertheless, it has to be taken into account that the routine use of ROTEM is limited by several factors, as devices are not routinely available on all ICUs, conduct of measurements is time consuming for ICU personnel and adds to the costs.

## Conclusion

Monitoring of alternative anticoagulation with argatroban or lepirudin in critically ill patients with heparin- induced thrombocytopenia or suspect of heparin-induced thrombocytopenia by aPTT is not ideal, as it does not correlate with plasma levels. In contrast, thrombin time and ROTEM CT better reflects plasma levels. In order to provide safe and effective anticoagulation in critically ill patients with need for alternative anticoagulation, the adequate use of thrombin time and ROTEM CT should be studied and established.

## Additional file

Additional file 1: Correlation between PT (Quick) and argatroban and lepirudin plasma levels. Description: PT (Quick) did not correlate with argatroban or lepirudin plasma levels. (DOC 95 kb)

## Abbreviations

aPTT: Activated partial thromboplastin time
CFT: Clot formation time
CT: Clotting time
EXTEM: Rotational thromboelastometry assay measuring extrinsic coagulation pathway
FIBTEM: Rotational thromboelastometry assay measuring fibrinogen status of the coagulation system
INTEM: Rotational thromboelastometry assay measuring intrinsic coagulation pathway
MCF: Maximum clot firmness
PT: Prothrombin time, German “Quick”
ROTEM: Rotational thromboelastometry
SAPS: Simplified acute physiology score
SOFA: Sequential organ failure assessment
TT: Thrombin time
