# Supplementary material for: Monitoring of argatroban and lepirudin anticoagulation in critically ill patients by conventional laboratory parameters and rotational thromboelastometry – a prospectively controlled randomized double-blind clinical trial
Source: BMC Anesthesiol. 2018 Feb 9;18:18. doi: 10.1186/s12871-018-0475-y (PMC5810183; doi:10.1186/s12871-018-0475-y)
Supplement: Supplementary file 1 — Correlation between PT (Quick) and argatroban and lepirudin plasma levels. Description: PT (Quick) did not correlate with argatroban or lepirudin plasma levels. (DOC 95 kb) [file 12871_2018_475_MOESM1_ESM.doc]

**Monitoring of argatroban and lepirudin anticoagulation in critically ill patients by conventional laboratory parameters and rotational thromboelastometry – a prospectively controlled randomized double-blind clinical trial**

Martin Beiderlinden, Patrick Werner, Astrid Bahlmann, Johann Kemper, Tobias Brezina, Maximilian Schäfer, Klaus Görlinger, Holger Seidel, Peter Kienbaum and Tanja A Treschan

**Additional file 1.** Correlation between PT (Quick) and argatroban and lepirudin plasma levels

X-axis depicts plasma concentration in µg/ml, Y-axis displays Quick in seconds. Each dot represents one pair of measurements. a) Argatroban, b) Lepirudin. Spearman correlation coefficient (r) and level of significance are presented in each panel, p<0.01 was considered statistically significant.


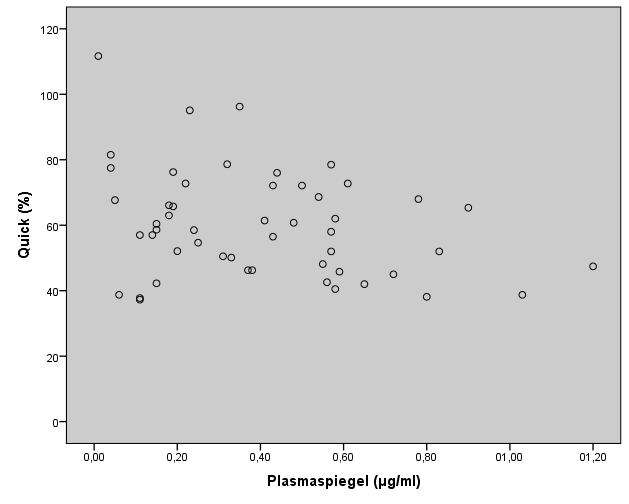


a)

r=-0.23; p=0.1


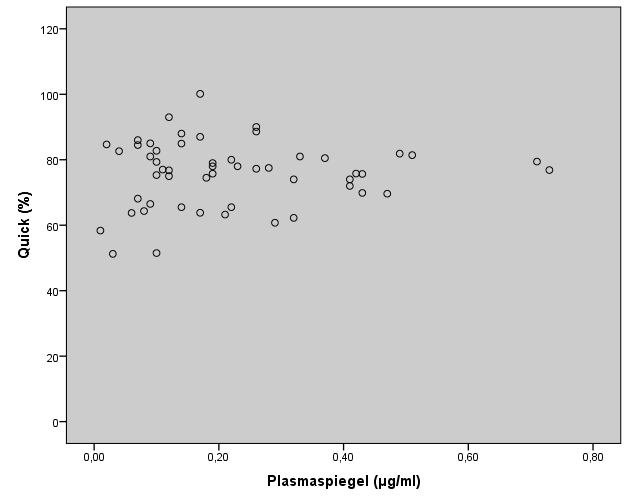


b)

r=0.05, p=0.97
